# Suppression of Neuronal Firing Following Antidromic High-Frequency Stimulations on the Neuronal Axons in Rat Hippocampal CA1 Region

**DOI:** 10.3389/fnins.2022.881426

**Published:** 2022-06-10

**Authors:** Yue Yuan, Zhouyan Feng, Gangsheng Yang, Xiangyu Ye, Zhaoxiang Wang

**Affiliations:** Key Lab of Biomedical Engineering for Education Ministry, College of Biomedical Engineering and Instrumentation Science, Zhejiang University, Hangzhou, China

**Keywords:** high-frequency stimulation, silent period, suppression, neuronal firing, evoked potentials, hippocampal CA1 region

## Abstract

High-frequency stimulation (HFS) of electrical pulses has been used to treat certain neurological diseases in brain with commonly utilized effects within stimulation periods. Post-stimulation effects after the end of HFS may also have functions but are lack of attention. To investigate the post-stimulation effects of HFS, we performed experiments in the rat hippocampal CA1 region *in vivo*. Sequences of 1-min antidromic-HFS (A-HFS) were applied at the alveus fibers. To evaluate the excitability of the neurons, separated orthodromic-tests (O-test) of paired pulses were applied at the Schaffer collaterals in the period of baseline, during late period of A-HFS, and following A-HFS. The evoked potentials of A-HFS pulses and O-test pulses were recorded at the stratum pyramidale and the stratum radiatum of CA1 region by an electrode array. The results showed that the antidromic population spikes (APS) evoked by the A-HFS pulses persisted through the entire 1-min period of 100 Hz A-HFS, though the APS amplitudes decreased significantly from the initial value of 9.9 ± 3.3 mV to the end value of 1.6 ± 0.60 mV. However, following the cessation of A-HFS, a silent period without neuronal firing appeared before the firing gradually recovered to the baseline level. The mean lengths of both silent period and recovery period of pyramidal cells (21.9 ± 22.9 and 172.8 ± 91.6 s) were significantly longer than those of interneurons (11.2 ± 8.9 and 45.6 ± 35.9 s). Furthermore, the orthodromic population spikes (OPS) and the field excitatory postsynaptic potentials (fEPSP) evoked by O-tests at ∼15 s following A-HFS decreased significantly, indicating the excitability of pyramidal cells decreased. In addition, when the pulse frequency of A-HFS was increased to 200, 400, and 800 Hz, the suppression of neuronal activity following A-HFS decreased rather than increased. These results indicated that the neurons with axons directly under HFS can generate a post-stimulation suppression of their excitability that may be due to an antidromic invasion of axonal A-HFS to somata and dendrites. The finding provides new clues to utilize post-stimulation effects generated in the intervals to design intermittent stimulations, such as closed-loop or adaptive stimulations.

## Introduction

Extracellular stimulations of electrical pulses in brain, commonly termed as deep brain stimulation (DBS), have been successfully used to treat movement disorders, such as Parkinson’s disease, essential tremor, and dystonia (Lee et al., 2019; Lozano et al., 2019). The therapy has been conventionally utilizing the actions of electrical pulses during stimulation periods, while the potential effects of DBS in the post-stimulation period have been lack of attention. However, some reports have shown that a relief of symptoms during DBS can last for a while after the cessation of stimulations, indicating an action of post-stimulation effects (Temperli et al., 2003). Furthermore, recent studies have shown that certain paradigms of DBS (e.g., a type of burst stimulations) can generate therapeutic efficiency lasting for hours after stimulations (Spix Teresa et al., 2021). Investigating post-stimulation effects is an important direction of DBS developments for saving electrical power and reducing risks, as well as for designing new stimulation paradigms used in intermittent stimulations such as adaptive DBS and closed-loop DBS.

DBS usually utilizes high-frequency stimulation (HFS) of pulse sequences around 100 Hz. Previous studies have reported that after the end of HFS, the neuronal excitability may experience a period of suppression. For example, in the primary motor cortex and subthalamic nucleus (STN) of rats, a marked decrease of excitatory postsynaptic current appeared after HFS (Iremonger et al., 2006; Shen and Johnson, 2008). Stimulations of the internal segment of globus pallidus (GPi) generated a decrease of neuronal firing in the post-stimulation period in the projection area of motor thalamus (Muralidharan et al., 2017). In addition, in human GPi and in rat hippocampus, the neuronal firing could completely disappear for seconds immediately following the cessation of HFS (Lafreniere-Roula et al., 2010; Feng et al., 2017; Wang et al., 2018). These reports have indicated that post-stimulation suppressions of neuronal activity could be common in brain regions. Furthermore, a clinic study of DBS on Parkinson’s disease reported that patients with a longer silent period of neuronal firing after 100 Hz stimulation in STN tended to obtain a better clinical outcome after DBS (Milosevic et al., 2017), which indicated that the post-stimulation suppression is of clinical significance.

Several possible mechanisms could cause a decrease of neuronal firing following HFS. For the neurons in the post-synaptic projection area, an increase of inhibitory inputs (e.g., from GABAergic inhibitory synapses) has been shown as a mechanism for the neuronal inhibition in the DBS of STN and GPi (Liu et al., 2012; Chiken and Nambu, 2013). Also, due to HFS-caused failures in axonal conductions or/and in synaptic transmissions, a decrease of inputs in excitatory synapses can decrease the firing of downstream neurons in the projection area (Feng et al., 2013; Rosenbaum et al., 2014). In addition, for the neurons directly under stimulations, a decrease of neuronal excitability or an increase of firing threshold could induce a silent period of neuronal firing following HFS (Beurrier et al., 2001).

We proposed here a new hypothesis that without involving synaptic transmissions, a post-stimulation suppression of firing could generate in the neurons after HFS at their axons. To verify the hypothesis, taking advantage of the clear lamellar structures of hippocampal region in brain, we applied HFS of electrical pulses on the axonal tract of pyramidal cells (the alveus fibers), so-called antidromic-HFS (A-HFS), in the rat hippocampal CA1 region *in vivo*. The antidromically-evoked potentials and post-stimulation neuronal firing were recorded and analyzed to reveal the effects of A-HFS and the possible underlying mechanisms. The results of this study may shed light on the post-stimulation effects of HFS and provide information for developing new paradigms of DBS therapy.

## Materials and Methods

### Animal Surgery

**FIGURE 1:**
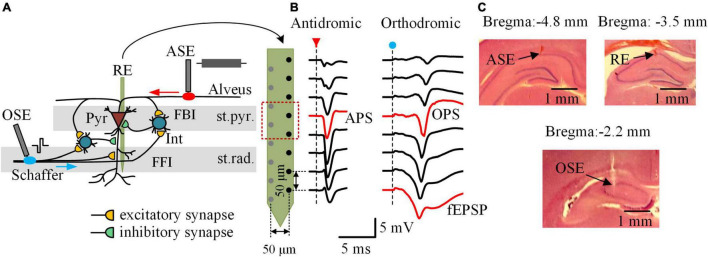
Local neuronal circuits, neuronal potentials, and electrode placements in the rat hippocampal CA1 region. **(A)** Schematic diagram of the local neuronal circuits and the electrode placements. The 16-channel recording electrode (RE) was placed across the stratum pyramidale (st.pyr.) and the stratum radiatum (st.rad.) of CA1 region. The antidromic-stimulation electrode (ASE) and the orthodromic-stimulation electrode (OSE) were located at the alveus fibers and the Schaffer collaterals, respectively. Interneurons (Int) constitute local circuits of feedforward inhibition (FFI) and feedback inhibition (FBI) acting on the pyramidal cells (Pyr), the principal neurons in CA1 region. **(B)** Waveforms of evoked potentials along the half channels of RE following an antidromic-pulse (red arrow) and an orthodromic-pulse (blue dot), respectively. The antidromic population spike (APS) and orthodromic population spike (OPS) recorded in the st.pyr. as well as the field excitatory postsynaptic potential (fEPSP) recorded in the st.rad. were denoted in red. **(C)** Example photographs of histological brain slices showing the electrode tracks of ASE, RE, and OSE in the coronal sections of ∼ 4.8, ∼3.5, and ∼2.2 mm posterior to bregma, respectively.

The protocol of animal experiment was approved by the Institutional Animal Care and Ethics Committee, Zhejiang University. Forty-eight adult male Sprague–Dawley rats were used from 8–12 weeks of age and weighted 323 ± 44 g in a range of 250–400 g. The rats were housed under a 12:12 h light-dark cycle with free access to food and water in a temperature- and humidity-controlled room. During the experiments, the rats were fixed in a stereotaxic apparatus (Stoelting Co., United States) under anesthesia after an intraperitoneal injection of urethane (1.25 g/kg). Details of the surgery and the electrode placements have been reported previously (Feng et al., 2014). In brief, a 16-channel array (#Poly2, NeuroNexus Technologies, United States) was used as recording electrode (RE) and inserted into the hippocampal CA1 region (AP −3.5 mm; ML 2.7 mm; DV ∼2.4 mm), across the stratum pyramidale (st.pyr.) and the stratum radiatum (st.rad.). Two concentric bipolar electrodes (#CBCSG75, FHC Inc., United States) were used as stimulation electrodes (ASE and OSE) and positioned respectively at the alveus fibers (AP −4.8 mm; ML 2.7 mm; DV ∼2.0 mm) and at the Schaffer collaterals (AP −2.2 mm; ML 2.1 mm; DV ∼2.8 mm). The ASE and OSE delivered electrical pulses to antidromically and orthodromically activate the neurons located near the RE (Figure 1A). The signals of multiple unit activity (MUA) as well as the waveforms of antidromic population spikes (APS), orthodromic population spikes (OPS), and field excitatory postsynaptic potentials (fEPSP) recorded along the RE array were used to justify the correct positions of the three electrodes (Figure 1B).

After the experiments, the rats were euthanized by intracardiac injection of 10% potassium chloride solution of lethal dose. The rat brain was then isolated and put into 4% paraformaldehyde overnight at 4°C. Brain slices were obtained and stained with hematoxyline-eosin to confirm the locations of the electrodes (Figure 1C).

### Stimulating and Recording

Stimuli were current pulses with a biphasic rectangle waveform and a width per phase of 0.1 ms. The pulses were generated by a stimulator (Model 3800, A-M Systems Inc., United States) and were delivered to the stimulation electrodes through stimulus isolators (Model 3820, A-M Systems Inc., United States). The current intensity of pulses was in a range of 0.3–0.5 mA that was able to induce an APS or OPS with an amplitude of approximately 75% of the maximal amplitude.

The duration of A-HFS was 1 min with a pulse frequency of 100, 200, 400, or 800 Hz. In a group of 40 rats, an A-HFS sequence of 100 Hz was performed once on each rat. In an additional group of 8 rats, A-HFS sequences of 100, 200, 400, and 800 Hz were performed in a random order on each rat. The interval between two adjacent A-HFS trains was longer than 30 min to ensure a recovery from the previous A-HFS. Separated test pulses with identical parameters as A-HFS pulses, termed as A-test, were applied in the baseline period before A-HFS and in the post-stimulation period after A-HFS to verify the recovery of neuronal activity.

In addition, in the eight rats performed A-HFS with four different frequencies, separated test pulses of orthodromic stimulations, termed as O-test, were applied at the Schaffer collaterals in the baseline period, during the 1-min A-HFS (at 45 and 55 s) and in the post-stimulation period after A-HFS to evaluate the excitability of pyramidal cells and the effect of local inhibitory circuits. The O-test was a pulse pair with an inter-pulse-interval of 50 ms that would induce OPS_1_ and OPS_2_ in the st.pyr. and induce fEPSP_1_ and fEPSP_2_ in the st.rad., respectively.

Extracellular potentials collected by the RE array were amplified 100 times by a 16-channel amplifier (Model 3600, A-M Systems Inc., United States) with a filtering range of 1–5,000 Hz. Then the amplified signals were sampled by a Powerlab data acquisition system (Model PL3516, ADInstruments Inc., Australia) at a rate of 20 kHz per channel and stored for off-line analyses.

### Data Analysis

Both APS and OPS were obtained from a recording channel located in the st.pyr. The fEPSP evoked by O-test pulses were recorded from a channel located in the st.rad., 200 μm below the OPS channel (Figure 1B). The following parameters of the evoked potentials were calculated by a custom-made MATLAB program. The APS amplitude was measured as the potential drop of the negative-going phase of its waveform, and the OPS amplitude was measured as the average potential of the negative- and positive-going phases of its waveform. The amplitude of APS and OPS can reflect the amount of neurons that synchronously fire action potentials following a pulse (Andersen et al., 1971; Varona et al., 2000). The absolute value of fEPSP slope was measured by a fitting line of seven sampling data using a least-square method around the maximum slope on the falling phase of the fEPSP waveform. A greater fEPSP slope means a greater amount of excitatory synaptic transmissions (Salmani et al., 2011). The amplitude ratio of OPS_2_/OPS_1_ and the slope ratio of fEPSP_2_/fEPSP_1_ of the paired-pulse O-test were calculated to evaluate the effect of the local inhibitory circuits. A smaller ratio indicates a greater inhibition from feedforward and feedback inhibitory circuits (Davies et al., 1990; Albertson et al., 1996). In addition, the changes of these indexes during and following the A-HFS were measured by their differences from the corresponding baseline levels.

To obtain MUA signals, stimulus artifacts in the raw signals were removed by replacing the artifact segments with short interpolation lines (Yu et al., 2015). Then, the signals were filtered by a digital high-pass filter with a cut-off frequency of 500 Hz. The MUA signals of four neighboring recording channels located in the st.pyr. of CA1 region were used for unit spike sorting (denoted by the red dashed box in Figure 1B). Feature vectors of the unit spikes (principal components and amplitudes) were calculated by a MATLAB program and were then used for spike sorting by an open-source software (SpikeSort 3D, Neuralynx Inc., www.Neuralynx.com). Unit spikes of interneurons (Int) and pyramidal cells (Pyr) were distinguished based on spike waveforms and their firing patterns in baseline. Unit spikes with a positive-going phase width <0.4 ms and with a regular firing pattern were classified as from interneurons, whereas those with a positive-going phase width >0.7 ms and with a burst firing pattern were classified as from pyramidal cells (Barthó et al., 2004). A total of 104 pyramidal cells and 66 interneurons were obtained in the 40 rats that performed 1-min 100 Hz A-HFS, with 2–3 pyramidal cells and 1–2 interneurons per rat.

Normalized by the mean firing rate in the 1-min baseline period, the normalized firing rate of each neuron was calculated with a time bin of 5 s in the periods of 1-min baseline before A-HFS and 4-min post-stimulation after A-HFS. Neurons with a baseline firing rate below 0.5 spikes/s had been excluded. The time distance from the end of A-HFS to the appearance of the first unit spike of a neuron after A-HFS was defined as the length of silent period of the neuron. And, the time distance from the end of A-HFS to the time when the firing rate of a neuron recovered to its mean baseline rate was defined as the length of recovery period of neuronal firing.

Statistical data were represented as mean ± standard deviation. Student *t*-test and one-way ANOVA with *post hoc* Bonferroni tests were used to judge the significance of differences among data groups. The relationship between the A-HFS frequency and a neuronal index, such as the length of silent period, the OPS_1_ amplitude, the fEPSP_1_ slope, the amplitude ratio OPS_2_/OPS_1_, and the slope ratio fEPSP_2_/fEPSP_1_, was described with a Pearson linear correlation. All the statistical analyses were fulfilled by SPSS Statistics 22 (IBM Inc., United States).

## Results

### Silent Period of Neuronal Firing Following Antidromic High-Frequency Stimulation

**FIGURE 2:**
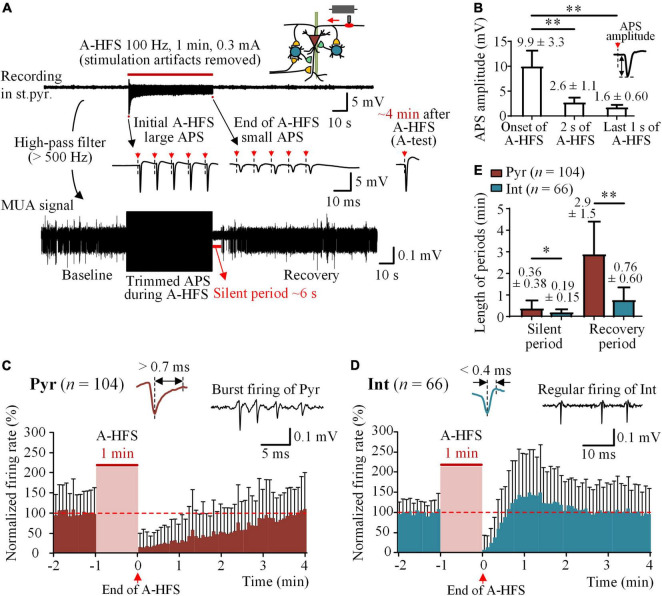
Silence of neuronal firing following A-HFS. **(A)** *Top*: a typical recording of neuronal potentials before, during, and after 1-min 100 Hz A-HFS in the stratum pyramidale (st.pyr.) of hippocampal CA1 region. A schematic diagram of stimulation and recording is shown on the upper-right corner. *Middle*: The expanded signals show the initial large APS and the end small APS evoked by the pulses of A-HFS. The red arrows with dashed lines denote the artifacts of pulse stimuli. *Bottom*: The MUA signal obtained by filtering the original recording shows the firing of unit spikes before and after A-HFS. **(B)** Comparisons of amplitudes of APSs evoked at the onset, 2 s, and the last 1 s of A-HFS. **P < 0.01, repeated one-way ANOVA with *post hoc* Bonferroni tests, *n* = 40 rats. **(C,D)** Time histograms of mean normalized firing rate of pyramidal cells (Pyr) and interneurons (Int) before and after A-HFS (time bin = 5 s). Typical spike waveforms and firing patterns of the two types of neurons are illustrated on the top. The red dashed lines denote the mean baseline firing rate, i.e., 100%. Because of the differences in the lengths of silent period of individual neurons, with some silences shorter than 5 s, a clear silent period with zero firing cannot appear in the histograms of mean firing rates of neurons with a bin of 5 s. **(E)** Comparisons of the lengths of silent period and recovery period following A-HFS between pyramidal cells and interneurons. *P < 0.05, **P < 0.01, unpaired *t*-test.

To investigate whether stimulations on axons can cause a post-stimulation suppression of firing on the neurons themselves under the stimulation, a 1-min train of 100 Hz A-HFS was applied at the alveus fibers, the axons of CA1 pyramidal cells (Figure 2A). Action potentials evoked at axons by an A-HFS pulse can propagate antidromically to the somata of pyramidal cells and induce the somata to fire action potentials synchronously to form an APS waveform. Similar to previous reports (Feng et al., 2013, 2014), during the initial period of A-HFS, the amplitude of evoked APS decreased rapidly and then maintained small till the end of A-HFS (Figure 2A, *top* and *middle*) due to the failures of neurons to follow each pulse of A-HFS to fire. The mean amplitude of the evoked APS significantly decreased from an initial value of 9.9 ± 3.3 to 2.6 ± 1.1 mV in 2 s (Figure 2B, *P* < 0.01, repeated one-way ANOVA with *post hoc* Bonferroni tests, *n* = 40 rats) and then slightly decreased to the end value of 1.6 ± 0.60 mV in the rest 58 s period of A-HFS. Although their amplitudes decreased, the consecutive small APSs in the late period of A-HFS indicated that the firing of pyramidal cells persisted through the entire period of A-HFS.

However, immediately following the end of A-HFS, a silent period of MUA with completely no neuronal firing appeared before the MUA gradually recovered to the baseline level (Figure 2A, *bottom*). To investigate the firing of individual neurons in the post-stimulation period, unit spikes of 104 pyramidal cells and 66 interneurons were obtained in 40 rat experiments with the 1-min 100 Hz A-HFS. In the 1-min baseline period before A-HFS, the mean firing rate of pyramidal cells was 4.8 ± 4.6 spikes/s and that of interneurons was 10.3 ± 9.4 spikes/s. The time histograms of normalized firing rates of the neurons showed the recovery period following the A-HFS (Figures 2C,D). The mean lengths of both the silent period and the recovery period of pyramidal cells were significantly longer than those of interneurons (Figure 2E, *P* < 0.05 for silent period, 21.9 ± 22.9 vs. 11.2 ± 8.9 s; *P* < 0.01 for recovery period, 2.9 ± 1.5 vs. 0.76 ± 0.60 min, unpaired *t*-test). Note that the time lengths were the statistical data of individual neurons and were different from the data of time histograms of all neurons shown in Figures 2C,D. The APS evoked by an A-test pulse recovered within 4 min following the end of A-HFS (Figure 2A, *right*). The recovery process of APS has been reported in our previous paper (Feng et al., 2014) and was omitted here.

These results showed a firing suppression for both pyramidal cells and interneurons in the post-stimulation period of axonal A-HFS. According to the constructure of local neuronal circuits of CA1 region illustrated in Figure 1A (Andersen et al., 2007), the firing suppression of pyramidal cells was a surprise because their afferent inputs should have not been affected by the axonal A-HFS at alveus, and a decrease of inhibitions due to the silence of interneurons should have facilitated rather than suppressed the firing of pyramidal cells. Therefore, a possible cause of the firing suppression could be a decrease of the excitability of pyramidal cells generated by the A-HFS. To verify the hypothesis, we next added O-test pulses at the afferent fibers, the Schaffer collaterals, to evaluate the changes of excitability of pyramidal cells.

### Decrease of the Excitability of Pyramidal Cells by Antidromic High-Frequency Stimulation

**FIGURE 3:**
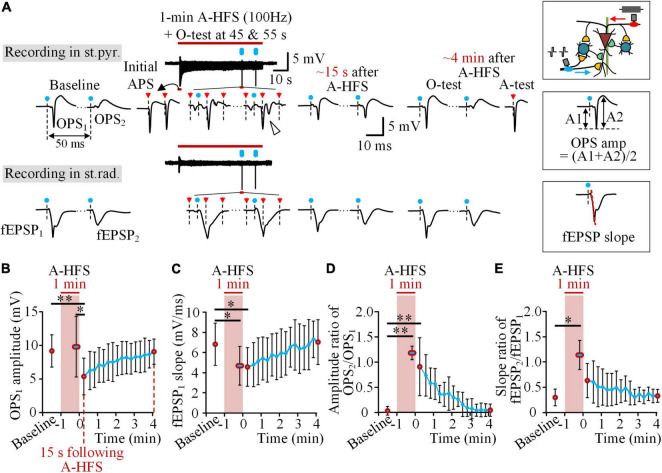
Decrease of the excitability of pyramidal cells following A-HFS. **(A)** Typical examples of OPS and fEPSP waveforms evoked by the O-test paired pulses before, during, and after 1-min 100 Hz A-HFS. During A-HFS, the O-tests were applied at 45 and 55 s of A-HFS. After A-HFS, the O-tests were applied every 15 s till ∼4 min after the end of A-HFS. The blue dots denote the artifacts of O-test paired pulses and the red arrows denote the artifacts of A-HFS pulses. A schematic diagram of stimulations and recordings is shown on the upper-right corner. The measurements of OPS amplitude and fEPSP slope are illustrated on the middle-right and bottom-right, respectively. **(B–E)** Changes of OPS_1_ amplitude **(B)**, fEPSP_1_ slope **(C)**, amplitude ratio of OPS_2_/OPS_1_ **(D)**, and slope ratio of fEPSP_2_/fEPSP_1_ **(E)** before (baseline), during, and after the 1-min 100 Hz A-HFS. Red circles denote the data of baseline, the average of two O-tests in late A-HFS, the data at 15 s following A-HFS, and the recovered data at ∼4 min after A-HFS. *P < 0.05, **P < 0.01, repeated one-way ANOVA with *post hoc* Bonferroni tests, *n* = 8 rats.

O-tests of paired pulses with a 50 ms interval were applied on the Schaffer collaterals to activate the pyramidal cells orthodromically in the baseline and in the post-stimulation period, as well as in the late period (45 and 55 s) of 1-min trains of 100 Hz A-HFS (Figure 3A). The evoked potentials of OPS_1_ and OPS_2_ in the st.pyr. as well as fEPSP_1_ and fEPSP_2_ in the st.rad. were analyzed. In baseline, OPS_2_ was much smaller than OPS_1_, indicating a paired-pulse depression (PPD) generated by inhibitions from local inhibitory circuits of interneurons (Davies et al., 1990; Albertson et al., 1996). In the late period of A-HFS, although the APS evoked by A-HFS pulses had already decreased to a fraction of its initial amplitude, O-tests were able to evoke large OPSs with some OPSs even including two spikes (denoted by the hollow-arrowhead in Figure 3A), indicating a high excitability of the neurons.

However, the evoked OPS decreased following the end of A-HFS. The mean amplitude of OPS_1_ evoked at the time 15 s following A-HFS was significantly smaller than those evoked in the periods of baseline and in the late A-HFS (Figure 3B, *P* < 0.01 or 0.05, repeated one-way ANOVA with *post hoc* Bonferroni tests, *n* = 8 rats), while the mean amplitude of OPS_1_ in the late A-HFS did not decrease. However, in the late A-HFS, the mean fEPSP_1_ slope had already decreased significantly (Figure 3C, *P* < 0.05, repeated one-way ANOVA with *post hoc* Bonferroni tests, *n* = 8 rats) and remained the decrease to the time 15 s following A-HFS. In addition, both the mean amplitude ratio of OPS_2_/OPS_1_ and the mean slope ratio of fEPSP_2_/fEPSP_1_ increased significantly in the late A-HFS (Figures 3D,E, *P* < 0.01 or 0.05, repeated one-way ANOVA with *post hoc* Bonferroni tests, *n* = 8 rats). The increases lasted to the post-stimulation period. The four indexes gradually returned to baseline levels in ∼4 min following the A-HFS (Figures 3B–E).

The increases of OPS_2_/OPS_1_ ratio and fEPSP_2_/fEPSP_1_ ratio again indicated a decrease of local inhibitions. Under this situation, both the decreases of OPS_1_ amplitude and fEPSP_1_ slope in the post-stimulation period indicated a decrease of neuronal excitability to respond to the orthodromic inputs from the Schaffer collaterals. In addition, the fEPSP_1_ slope had already decreased during A-HFS, indicating that the antidromic activations of A-HFS from axons to somata and even further to dendrites could affect the neuronal excitability. If so, the post-stimulation suppression would be attenuated by A-HFS with a higher pulse frequency, because a higher frequency could induce a deeper axonal blockage around the stimulation site, thereby resulting in the axons conducting less antidromic activations to affect the somata (Jensen and Durand, 2009; Zheng et al., 2011; Feng et al., 2013, 2014). Thus, we next applied A-HFS with a frequency higher than 100 Hz to see whether or not the post-stimulation suppressions would decrease.

### Neuronal Excitability Following Antidromic High-Frequency Stimulation With Different Pulse Frequencies

**FIGURE 4:**
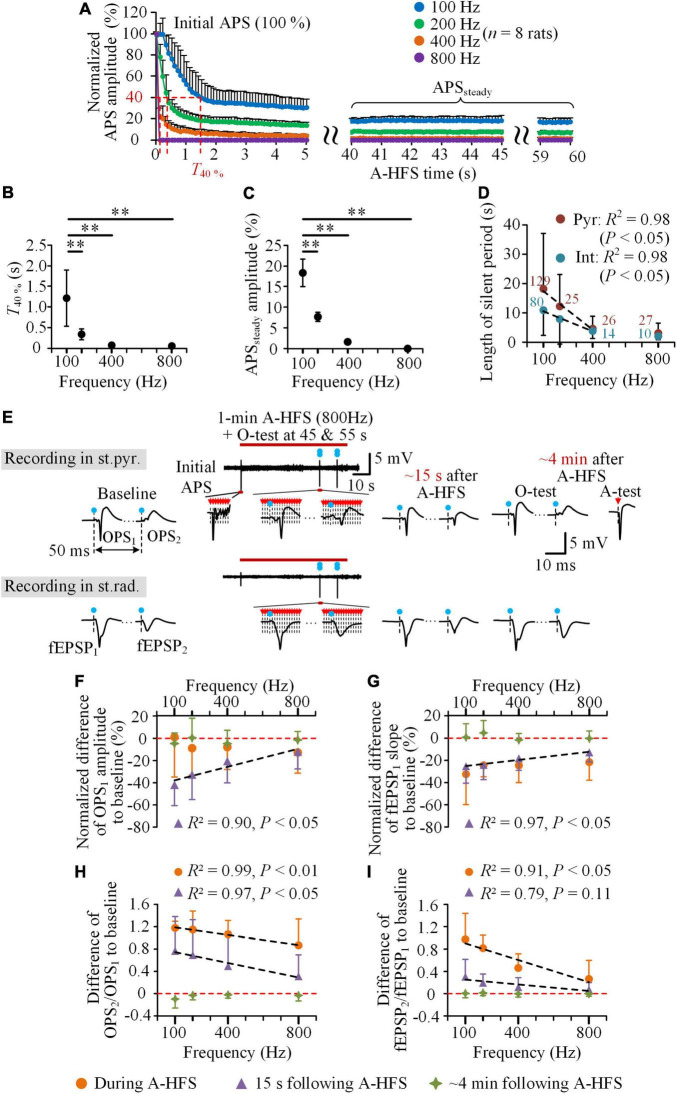
Neuronal responses to the trains of A-HFS with different pulse frequencies. **(A)** The mean normalized APS amplitudes evoked by each pulse during 1-min A-HFS with 100, 200, 400, and 800 Hz. Except the amplitude of the first APS being 100%, the other data were the average amplitude of APSs evoked by 10 successive pulses at every 0.1 s. **(B,C)** Changes of the time for the APS amplitude dropping to 40% (*T*_40%_) **(B)** and the average amplitude of APSs in the late 40–60 s period of A-HFS (APS_*steady*_) **(C)** with the A-HFS frequencies. **P < 0.01, one-way ANOVA with *post hoc* Bonferroni tests, *n* = 8 rats. **(D)** Changes of the lengths of silent period following the A-HFS with different frequencies for the two types of neurons (Pyr & Int). The digits are the number of individual neurons from 48 rats (100 Hz) and eight rats (200, 400, and 800 Hz). **(E)** Typical examples of the OPSs in the st.pyr. and fEPSPs in the st.rad. evoked by the O-test paired pulses before, during, and after 1-min 800 Hz A-HFS. The blue dots with dashed lines denote the artifacts of O-tests and the red arrows with dashed lines denote the artifacts of A-HFS pulses and A-test pulse. **(F–I)** Comparisons of normalized difference of OPS_1_ amplitude **(F)**, normalized difference of fEPSP_1_ slope **(G)**, difference of OPS_2_/OPS_1_ **(H)**, and difference of fEPSP_2_/fEPSP_1_ **(I)** to baseline during A-HFS, at 15 s following and at ∼4 min after the end of A-HFS with different frequencies (*n* = 8 rats). Negative values mean decreases and positive values mean increases from baselines denoted by the red dashed lines. The black dashed lines in **(D)** and **(F–I)** denote Pearson linear correlations between the abscissa and ordinate values with the results of *R*^2^ and *P* shown on the upside or downside.

During the periods of 1-min A-HFS of 100, 200, 400, and 800 Hz, with a higher frequency, the evoked APS decreased faster at the initial period and was suppressed more in the late period (Figure 4A). From the same initial normalized APS amplitudes (100%), the time for the APS amplitude dropping to 40% (*T*_40%_) was 1.2 ± 0.7 s for the 100 Hz A-HFS and significantly decreased to only 0.06 ± 0.0005 s for the 800 Hz A-HFS (Figure 4B, *P* < 0.01, one-way ANOVA with *post hoc* Bonferroni tests, *n* = 8 rats). In addition, in the late 20 s period (40–60 s) of A-HFS, the normalized amplitude of steady APS (APS_*steady*_) was 18.4 ± 3.4% for 100 Hz A-HFS and decreased to a disappearance (0) for 800 Hz A-HFS (Figure 4C, *P* < 0.01, one-way ANOVA with *post hoc* Bonferroni tests, *n* = 8 rats). These changes of APS indicated a faster and deeper axonal block induced by a higher pulse frequency, which was consistent with previous reports (Feng et al., 2013, 2014).

In addition, the mean length of silent period of unit firing following the A-HFS decreased significantly with the increase of the pulse frequency (Figure 4D). The silent period of pyramidal cells decreased from 18.1 ± 18.8 s for 100 Hz A-HFS to only 4.6 ± 4.2 s for 400 Hz and 3.1 ± 3.4 s for 800 Hz A-HFS, while the silent period of interneurons decreased from 10.9 ± 8.5 s for 100 Hz A-HFS to only 3.8 ± 2.5 s for 400 Hz and 2.1 ± 1.2 s for 800 Hz A-HFS. The results of Pearson linear correlations showed a significant correlation between the mean length of silent periods and the frequencies in 100–400 Hz for both pyramidal cells and interneurons (Figure 4D, *R*^2^ = 0.98, *P* < 0.05).

To evaluate the decrease of excitability of pyramidal cells, the same O-tests of paired pulses were applied in the baseline and in the post-stimulation period as well as in the late period (45 and 55 s) of A-HFS with the four different frequencies. The evoked potentials of O-tests (OPSs and fEPSPs) changed less during and following A-HFS with a higher pulse frequency, e.g., 800 Hz (Figure 4E). Normalized by the baseline value, the mean normalized OPS_1_ amplitude at 15 s following 800 Hz A-HFS decreased less than 15%. This decrease of OPS_1_ was substantially smaller than the corresponding decrease following 100 Hz A-HFS (∼40%, Figure 4F), so did the decrease of fEPSP_1_ slope (Figure 4G). These decreases of OPS_1_ amplitude and fEPSP_1_ slope correlated significantly with the frequencies of A-HFS: the smaller the frequency, the greater the decrease (Figures 4F,G, *R*^2^ > 0.90, *P* < 0.05). The decreases of the two indexes during A-HFS were smaller than or similar to the values at 15 s following A-HFS. The decreases at ∼4 min after A-HFS approached zero, indicating a recovery to the baseline level.

In addition, the increases of amplitude ratio of OPS_2_/OPS_1_ from baseline levels both to the late A-HFS and to 15 s following A-HFS correlated significantly with the frequencies of A-HFS: smaller the frequency, greater the increase (Figure 4H, *R*^2^ > 0.97, *P* < 0.01 or 0.05). The increases of slope ratio of fEPSP_2_/fEPSP_1_ also presented a similar trend (Figure 4I).

These results indicated that the post-stimulation effects, including the silence of neuronal firing, the decreases of orthodromically evoked potentials (OPS_1_ and fEPSP_1_), and the increases of ratios (OPS_2_/OPS_1_ and fEPSP_2_/fEPSP_1_), were all related with the pulse frequency of A-HFS, especially in the frequency range of 100–400 Hz.

## Discussion

The major findings of the study include: (1) the excitability of pyramidal cells was decreased following 1-min 100 Hz A-HFS on their axons thereby causing a silent period for seconds and a suppressed period for minutes in the post-stimulation neuronal firing in the rat hippocampal CA1 region. (2) A-HFS with a higher frequency did not increase but decrease the post-stimulation suppression of neuronal firing. The possible underlying mechanisms for the post-stimulation suppression are analyzed below.

### Possible Mechanisms Underlying the Suppression of Neuronal Firing Following Axonal Stimulations

The first interesting finding is that the neurons, especially the pyramidal cells (the principal neurons of CA1), stopped firing for a while following A-HFS on their axons, instead of continuing the firing of A-HFS period or returning to the baseline firing. The firing suppression could be caused by changes in pre-synaptic inputs, such as an increase of inhibitory inputs and/or a decrease of excitatory inputs, and/or by changes in post-synaptic side, such as a decrease of excitability of the neurons themselves. Their possibilities are analyzed below.

Previous studies have shown inhibitions of neuronal firing following the DBS of STN and GPi in patients and rats. Those inhibitions have been considered resulting from the activation of GABAergic synapses by the stimulations thereby increasing the inhibitory inputs to the neurons (Dostrovsky et al., 2000; Wu et al., 2001; Lafreniere-Roula et al., 2010). However, those inhibitions can only last a fraction of a second. Here, in our results, the silent periods of pyramidal cells can last for tens of seconds beyond the stimulations. Although the pulses of A-HFS could have activated the interneurons in the feedback inhibitory circuits to in turn inhibit the pyramidal cells through GABAergic synapses (Knowles and Schwartzkroin, 1981; Lacaille et al., 1987; Pelkey et al., 2017), the activation of inhibitory synapses by the stimulations could not last for such a long time. The interneurons were silent themselves after the end of A-HFS (Figures 2D,E), indicating a lack of continuous inhibitions from local inhibitory circuits as illustrated in Figure 1A. In addition, the increase of OPS_2_/OPS_1_ evoked by paired pulses also indicated a decrease rather than an increase of inhibitions (Figure 3D). Therefore, an increase of inhibitions cannot be a mechanism for the post-stimulation suppression. Neither can a decrease of pre-synaptic excitatory inputs from afferent fibers, because the A-HFS at alveus, the efferent fibers, should not affect the afferent fibers substantially (Figure 1A). The unbalanced decrease of inhibitions should have increased the neuronal firing instead of suppression. Thus, the firing suppression of pyramidal cells should be due to a decrease of excitability of the neurons themselves.

The decrease of fEPSP_1_ slopes during A-HFS and in post-stimulation periods (Figure 3C), as well as the decrease of OPS_1_ amplitudes in post-stimulation periods (Figure 3B), indicated a decrease of the excitability of pyramidal cells. Previous reports have shown that action potentials (AP) evoked by pulses at axons (the alveus) of pyramidal cells can travel antidromically to their somata and then to their dendrites by a way of back-propagation. The back-propagation is mediated by sodium (Na^+^) channels in dendrites (Miyakawa and Kato, 1986). During repeated axonal activations, the back-propagation AP to dendrites can decrease rapidly due to an inactivation of persistent Na^+^ channels that are essential for dendrite activations (Colbert et al., 1997; Beurrier et al., 2001). The decrease of dendrite excitability can hinder the dendrites to accept and spread the inputs from excitatory synapses thereby decreasing the excitability of pyramidal cells (Lipowsky et al., 1996; Canals et al., 2005). Only after a recovery of dendritic Na^+^ channels can the trans-synaptic inputs once again elicit neuronal firing. These mechanisms can explain the decrease of fEPSP_1_ slopes in our results. However, the decrease of OPS_1_ amplitudes appeared after A-HFS, not during A-HFS. The maintenance of OPS_1_ during A-HFS (Figure 3B) may be due to a sustenance of the excitability in somata by the continuous inputs of antidromic activations from A-HFS.

The second interesting finding is that in the pulse frequency range 100–800 Hz, A-HFS at axons with a higher frequency can generate a weaker post-stimulation effect rather than a stronger one in the stimulated pyramidal cells, indicating a less decrease of neuronal excitability caused by a higher frequency. Previous studies have shown that continuous activations at axons by A-HFS pulses can ultimately bring the axons to a sustained depolarization state, the so-called axonal block (Jensen and Durand, 2009; Zheng et al., 2011; Feng et al., 2013). Under the situation, the axons cannot follow each pulse to fire an action potential but can only intermittently follow a part of pulses (Guo et al., 2018). A-HFS with a higher frequency can generate a deeper axonal block, thereby permitting less action potentials to successfully propagate to somata and to dendrites to affect the excitability of neurons. The faster and more APS suppression by A-HFS with a higher frequency indicated a deeper axonal block and less amount of action potentials evoked in the cell bodies of pyramidal cells by each pulse (Figures 4A–C). Note that an APS waveform is formed mainly by action potentials of pyramidal cells because of their high dense distribution in the st.pyr. (Andersen et al., 2007). Therefore, the higher the pulse frequency, the less the post-stimulation effects were, indicated by a shorter silent period (Figure 4D) and smaller alterations in the responses to orthodromic activation inputs (i.e., the inputs from O-tests, Figures 4F–I).

In addition, the interneurons in the local circuits are activated by pyramidal cells through excitatory synaptic transmissions (Figure 1A). The post-stimulation suppression of interneuron firing may be due to a transient loss of excitatory inputs from the silenced pyramidal cells. After A-HFS, because the interneurons have a lower threshold of action potential generation than pyramidal cells (Csicsvari et al., 1998), the firing of interneurons recovered earlier and faster than pyramidal cells (Figures 2C,D), which could in turn inhibit the pyramidal cells and prolong the suppression period of pyramidal cells. Furthermore, pyramidal cells may connect to each other through excitatory synapses locally (Andersen et al., 2007). A lack of these excitatory inputs due to the silence of pyramidal cells may also result in a longer suppression period of pyramidal cells themselves. Nevertheless, CA1 pyramidal cells do not have extensive interconnections (Knowles and Schwartzkroin, 1981). Therefore, a lack of inter-excitations may not be a major contribution to the post-stimulation suppression of pyramidal cells.

Taken together, a decrease of excitability induced by an antidromic invasion of the effect of axonal A-HFS to somata and dendrites of pyramidal cells may generate the post-stimulation suppression of their firing. Stimulations with a higher pulse frequency may induce a deeper axonal block and generate less antidromic invasions thereby decreasing the post-stimulation effects. Nevertheless, further studies are needed to reveal more evidence to support these putative mechanisms.

### Implications and Limitations

By utilizing the specific lamellar structure of hippocampus, here we firstly show that HFS at axons can induce a post-stimulation suppression of neuronal firing. The suppression appearing on the neurons under axonal HFS was different from the neuronal suppressions appearing in the post-synaptic regions downstream of the stimulation site that have been reported mostly in previous studies (Lafreniere-Roula et al., 2009; Lafreniere-Roula et al., 2010; Liu et al., 2012; Chiken and Nambu, 2013; Feng et al., 2017; Wang et al., 2018). Furthermore, our finding suggests that besides other possible mechanisms, the suppressions of neuronal firing in the downstream may be caused by a firing stop of the pre-synaptic neurons with their axons under stimulations. According to previous reports (Feng et al., 2017; Wang et al., 2018), the suppression periods of post-synaptic neurons in the downstream region or distant projection region are longer than the suppressions of neurons immediately under stimulations presented here. It may be caused by the fact that the post-synaptic neurons can only gradually recover their firing after the recovery of excitatory inputs from pre-synaptic neurons. Therefore, the involvement of synapses may enlarge the suppression effect of axonal HFS.

Furthermore, the suppression or silence of neuronal firing induced by HFS can act as a destroy-like effect at the stimulation site to block the information flow in neuronal circuits (Lauritzen and Strong, 2016). Generally, HFS trains of a higher pulse frequency with a greater electrical energy can make a deeper degree of real neuronal damage. However, the axonal HFS with a lower pulse frequency, such as 100 Hz (in the commonly used frequency range of DBS), can generate a longer period of neuronal suppression than the HFS with a higher frequency of 200–800 Hz. This finding provides new information for developing DBS paradigms by utilizing post-stimulation effects. Especially for closed-loop stimulations that need to switch between on and off states frequently, the post-stimulation effects in the intervals of adjacent stimulations could play an important role. For instance, the suppression of neuronal firing may prevent the propagation of abnormal neuronal activity associated with disorders that otherwise could reappear in the intervals. Previous studies have shown that DBS with pauses can be as effective as continuous DBS (Kuncel et al., 2012; Swan et al., 2016). Our study suggested that post-stimulation suppressions could be an underlying mechanism.

In addition, axons are everywhere in brain, and the axon has the lowest threshold in all of the neuronal elements to respond to the narrow electrical pulses of DBS. Therefore, the activation of axons plays an important role in DBS therapy (Gradinaru et al., 2009; Grahn et al., 2014). Our present study revealed a new phenomenon of axonal HFS-induced suppression of neuronal firing and its putative mechanism, which provides new clues for advancing DBS applications.

Nevertheless, further studies are needed to verify the universality of the post-stimulation effects in brain regions other than hippocampus. In addition, the exact underlying mechanisms need to be revealed by more investigations. Moreover, appropriate stimulation paradigms need to be established to utilize the post-stimulation effects for clinical applications.

## Conclusion

The study first shows that sustained axonal HFS can decrease the excitability of neurons directly under the stimulation, thereby generating a suppression period of neuronal firing after the cessation of the stimulation. The suppression effect of HFS in the post-stimulation period provides important information for the development of new DBS paradigms, especially for the investigations of closed-loop DBS.

## Data Availability Statement

The original contributions presented in this study are included in the article/supplementary material, further inquiries can be directed to the corresponding author/s.

## Ethics Statement

The animal study was reviewed and approved by the Institutional Animal Care and Ethics Committee, Zhejiang University.

## Author Contributions

ZF and YY conceived and designed the study. YY, GY, and XY performed the animal experiments and analyzed the experimental data. YY, ZF, and ZW interpreted the results and wrote the manuscript. All authors approved the final version for submission.

## Conflict of Interest

The authors declare that the research was conducted in the absence of any commercial or financial relationships that could be construed as a potential conflict of interest.

## Publisher’s Note

All claims expressed in this article are solely those of the authors and do not necessarily represent those of their affiliated organizations, or those of the publisher, the editors and the reviewers. Any product that may be evaluated in this article, or claim that may be made by its manufacturer, is not guaranteed or endorsed by the publisher.
